# A Speech Analytics-Based Methodological Protocol for Monitoring Orthopedic Rehabilitation in the Brazilian Unified Health System

**DOI:** 10.3390/ijerph23050626

**Published:** 2026-05-08

**Authors:** Rafael Baena Neto, Vicente Idalberto Becerra Sablón

**Affiliations:** Health Data Science Postgraduate Program, São Francisco University (USF), Bragança Paulista 12916-900, SP, Brazil; vicente.sablon@usf.edu.br

**Keywords:** speech analytics, digital health, orthopedic rehabilitation, public health, health data analytics, clinical communication, Brazilian Unified Health System, SUS

## Abstract

**Highlights:**

**Public health relevance—How does this work relate to a public health issue?**

- Unstructured clinical conversations play a central role in orthopedic rehabilitation within public health systems; however, they remain underused as analyzable sources for monitoring therapeutic communication and rehabilitation trajectories in the Brazilian Unified Health System (SUS).
- Speech analytics offers a promising methodological basis for transforming these interactions into structured analytical representations that may support future population-level monitoring of musculoskeletal rehabilitation processes.

**Public health significance—Why is this work of significance to public health?**

- This study presents a prospective methodological protocol, rather than a validated clinical system, for the ethical and interoperable integration of speech-based data into public health rehabilitation workflows.
- The proposed framework combines automatic speech recognition (ASR), speaker diarization, semantic analysis, and knowledge-grounding components within a single evaluative architecture designed for future empirical validation in SUS settings.

**Public health implications—What are the key implications or messages for practitioners, policy makers and/or researchers in public health?**

- The protocol is intended to support future studies on communication-derived indicators related to treatment adherence, patient-reported difficulties, and rehabilitation follow-up.
- For researchers and policymakers, the study provides a methodological foundation for subsequent empirical investigations integrating speech analytics into public health rehabilitation systems.

**Abstract:**

The digital transformation of health systems and the increasing adoption of data-driven public health strategies have intensified the need for methods capable of capturing, structuring, and analyzing information derived from clinical interactions. In the Brazilian Unified Health System (SUS), orthopedic rehabilitation and therapeutic exercise prescription rely heavily on communication between healthcare professionals and patients, particularly with regard to understanding instructions, reporting symptoms, and identifying barriers to treatment continuity. However, much of this information remains embedded in unstructured spoken interactions, limiting its use for monitoring and evaluation purposes. This study presents a prospective methodological protocol for the future development and validation of a speech analytics architecture designed to analyze verbal interactions in orthopedic rehabilitation within the SUS. The proposed framework integrates automatic speech recognition, speaker diarization, semantic processing with large language models (LLMs), biomedical entity extraction, and retrieval-grounded analytical components to generate structured indicators from clinical speech. In addition, the manuscript includes an illustrative simulation based on administrative proxy data converted into synthetic narratives in order to exemplify the expected structure of downstream analytical outputs. This simulation does not constitute validation of the full audio-based pipeline, but rather serves to clarify the proposed analytical workflow. Overall, the protocol establishes a structured methodological basis for future empirical studies aimed at evaluating the technical performance, semantic validity, and potential public health utility of speech analytics in rehabilitation monitoring, under appropriate ethical, regulatory, and data protection safeguards.

## 1. Introduction

Musculoskeletal conditions affect approximately 1.71 billion people worldwide, making them the largest contributor to years lived with disability globally. Global epidemiological analyses indicate that conditions affecting the musculoskeletal system account for a substantial proportion of years lived with disability and generate significant demand for rehabilitation services across health systems [1,2].

Orthopedic rehabilitation therefore plays a central role in restoring functional capacity, reducing pain, and improving quality of life for affected individuals. However, the effectiveness of rehabilitation programs depends not only on clinical interventions, but also on patient adherence to prescribed therapeutic exercises and recommendations. Previous studies have shown that barriers related to motivation, communication, and therapeutic engagement frequently influence adherence in musculoskeletal rehabilitation contexts [3,4]. In musculoskeletal specialty care, patient-rated physician communication effectiveness and satisfaction have also been associated with age and psychological factors, reinforcing the clinical relevance of communication-sensitive monitoring strategies in orthopedic settings [5,6].

In many healthcare environments—particularly in public health systems—monitoring adherence and patient engagement remains challenging. Clinical follow-up often relies on self-reported information and short consultation times, which limits the systematic observation of communication patterns, patient concerns, and barriers that may affect treatment continuity during routine care interactions [7,8].

Recent advances in digital health and artificial intelligence have opened new possibilities for analyzing healthcare communication. In particular, speech analytics technologies that combine automatic speech recognition, natural language processing, and large language models enable the structured analysis of verbal interactions between patients and healthcare professionals [9,10,11]. These approaches have been increasingly explored in domains such as telemedicine, clinical documentation support, and healthcare service monitoring [10,11,12].

Despite these technological advances, most current applications remain focused on improving documentation workflows or supporting clinical note generation. Fewer studies have investigated how verbal interactions themselves can be systematically analyzed to generate indicators relevant to treatment adherence, therapeutic engagement, and rehabilitation monitoring in real-world healthcare systems [9,10,11].

Consequently, there remains a methodological gap in frameworks capable of integrating speech processing, semantic interpretation, and clinically meaningful monitoring indicators within public healthcare environments.

Despite these advances, there remains a methodological and evaluative gap in frameworks capable of integrating speech processing, semantic interpretation, and clinically relevant monitoring indicators within public healthcare environments such as the SUS. In particular, the literature still lacks prospective protocols that clearly define how speech-derived indicators should be structured, interpreted, and subsequently validated in real rehabilitation settings.

This study proposes a prospective methodological protocol for the future development and validation of a speech analytics architecture designed to analyze verbal interactions in orthopedic rehabilitation within the SUS. The proposed framework integrates automatic speech recognition, speaker diarization, semantic processing, biomedical entity extraction, semantic indexing, and retrieval-grounded analysis within a single methodological architecture intended for future empirical assessment. As an illustrative complement, the manuscript also presents a proxy-data simulation based on structured administrative records converted into synthetic narratives in order to exemplify the expected organization of analytical outputs. Accordingly, the present contribution is methodological: it defines an integrated protocol, clarifies its evaluative trajectory, and provides an auditable illustration of the intended downstream analytical structure without claiming validation of the full real-audio pipeline at this stage.

## 2. Background and Related Work

Orthopedic rehabilitation and therapeutic exercise are inherently longitudinal processes that depend strongly on patient adherence and effective clinical communication. Studies in public health and musculoskeletal rehabilitation consistently show that communication failures and low adherence negatively affect clinical outcomes, increasing treatment duration, healthcare costs, and the risk of persistent functional disability, particularly in large-scale public health systems such as the SUS [7,13].

At the same time, digital health strategies have been widely discussed as mechanisms to improve care quality, promote interoperability, and enhance data-driven clinical and managerial decision-making [8,14,15]. Despite advances in electronic health records and health information systems, a substantial portion of clinical data remains unstructured, particularly information derived from verbal interactions between healthcare professionals and patients. This limitation restricts the analytical use of such data for care monitoring and public policy evaluation [15,16].

Speech analytics has emerged as a promising approach for transforming spoken data into structured and analyzable information, enabling the assessment of therapeutic communication quality, monitoring of treatment adherence, and longitudinal tracking of clinical evolution [9,12].

The integration of these techniques with LLMs further expands the semantic interpretation capabilities, moving beyond keyword-based approaches and enabling deeper analysis of clinical discourse in digital health environments [17,18,19].

However, the literature also highlights relevant limitations associated with the isolated use of generative models, particularly regarding reliability, traceability, and safety in clinical applications [19,20]. RAG approaches have been proposed to mitigate these risks by grounding generated outputs in trusted external sources, such as clinical guidelines, therapeutic protocols, and updated scientific literature [21,22,23,24]. Recent health-system perspectives further argue that RAG may also contribute to transparency and equity by retrieving evidence relevant to underrepresented populations, although these gains remain dependent on source quality, source validation, and privacy safeguards [25].

From a technical perspective, developments in semantic vectorization [26,27], biomedical named entity recognition [28,29,30,31], and analytical visualization [32,33] further support the contextualization and interpretation of clinically relevant discourse at scale [26,27,28,29,30,31,32,33,34]. Recent advances in ASR and speaker diarization have strengthened speech-based pipelines for analyzing verbal interactions in healthcare [35,36,37,38,39,40,41,42,43,44]. Models such as Whisper demonstrate strong generalization in real-world clinical settings [36], while modern diarization and natural language processing techniques enable role identification, extraction of relevant clinical entities, and contextualization of discourse within large-scale semantic representations [26,27,28,29,30,31,34,39,40,41,42,43,44].

Recent health-system perspectives further argue that RAG may also contribute to transparency and equity by retrieving evidence relevant to underrepresented populations, although these gains remain dependent on source quality, source validation, and privacy safeguards [25].

Despite these advances, there remains a lack of studies that systematically integrate speech analytics, LLMs, and RAG in applications specifically focused on orthopedic rehabilitation and therapeutic exercise within public health contexts. In particular, the literature reveals a gap in the operationalization of these technologies for large-scale, longitudinal monitoring of therapeutic communication, treatment adherence, and functional outcomes in public healthcare systems such as the SUS.

Therefore, this study contributes to the state-of-the-art by proposing an integrated approach for the automated analysis of verbal interactions in orthopedic rehabilitation, combining speech analytics, LLMs, and contextual knowledge retrieval. By aligning advanced technological foundations with real-world public health demands, the proposed protocol aims to support clinical decision-making, improve therapeutic follow-up, and strengthen care management and policy evaluation strategies in digital health.

### 2.1. LLMs

Recent evaluations have explored the application of LLMs in clinical reasoning, medical documentation, and biomedical knowledge extraction, demonstrating promising performance across several benchmark datasets [17,18,19].

These models represent a major recent advance in artificial intelligence, as they are trained on large-scale textual data to understand and generate natural language with high semantic and contextual coherence [17,20]. They have demonstrated strong capabilities in interpreting complex texts, performing contextual reasoning, and extracting meaning, making them particularly relevant for applications in digital health [17,18,19].

In healthcare settings, LLMs have been increasingly used to support the analysis of clinical records, electronic health records, and clinician-patient interactions, enabling the automated identification of symptoms, communicative intentions, and implicit clinical knowledge expressed in natural language [16,17,18,19]. These capabilities are especially important in orthopedic rehabilitation and human movement research, where patient-reported perceptions of pain, functional limitations, and therapeutic progress play a central role in clinical outcomes and influence the quality of documentation and follow-up care [3,4,5,6,7].

When integrated into speech analytics systems, LLMs extend traditional keyword-based approaches by enabling a deeper semantic interpretation of clinical conversations, as well as improved summarization and structuring of spoken content for care support [17,18,19]. This integration facilitates a longitudinal understanding of the therapeutic process and supports the monitoring of treatment adherence, therapeutic communication, and care quality, with potential benefits for clinical and operational decision-making in digital health contexts [5,6,7,15,44].

### 2.2. Retrieval-Augmented Generation

Retrieval-augmented generation (RAG) is an approach that combines generative language models with information retrieval mechanisms from external knowledge sources, allowing relevant documents to be consulted during response generation [21]. Unlike purely generative models, RAG enables access to structured documents, clinical guidelines, therapeutic protocols, and scientific literature at inference time, increasing the accuracy, traceability, and reliability in knowledge-intensive tasks [21].

In digital health, RAG has proven particularly valuable for reducing risks associated with imprecise or unsupported outputs by grounding interpretations in reliable and up-to-date sources, thereby enhancing safety and auditability in clinical applications [22,23]. In orthopedic rehabilitation and movement-related care, this approach enables patient-reported narratives to be systematically linked to clinical guidelines and scientific evidence, strengthening decision support and improving the consistency of clinical reasoning throughout the care process [22,24,25].

When integrated with speech analytics platforms, RAG enriches the analysis of verbal interactions by adding clinical and scientific context, supporting the identification of relevant patterns for screening, therapeutic follow-up, and chronic condition monitoring [22,23,25]. Within the SUS, this integration holds potential to support quality-of-life promotion, disease prevention, and resource optimization by transforming unstructured speech data into actionable information for care management and protocol evaluation [14,15,44].

### 2.3. Speech Analytics Enabling Technologies

#### 2.3.1. Automatic Speech Recognition and the Whisper Model

Automatic speech recognition (ASR) is the technology responsible for converting speech signals into text and represents the foundational step in speech analytics systems applied to healthcare [45,46]. In clinical settings, transcription quality directly affects the accuracy of medical records, the interpretation of patient narratives, and the reliability of subsequent natural language processing analyses [36,37,46].

Recent benchmarks indicate that modern transformer-based ASR systems demonstrate strong performance in multilingual and domain-specific scenarios, including Portuguese-language speech recognition and healthcare-related audio transcription [35,37,38].

Building on these advances, contemporary ASR models have achieved high accuracy even in challenging conditions, such as noisy environments, multiple speakers, and specialized domains, including medical language and musculoskeletal rehabilitation [35,36,37,38]. In digital health and telemedicine contexts, this robustness is particularly important, as remote consultations often involve acoustic variability, regional accents, and domain-specific terminology [14,36,38,39].

Earlier ASR approaches relied on smaller and more homogeneous audio-text datasets, which limited their ability to generalize across diverse clinical scenarios [47]. More recent self-supervised pretraining strategies improved performance but still faced constraints related to multilingual support and domain adaptation [36,48].

The Whisper model, developed by OpenAI, represents a significant advancement in ASR. Trained on a large and diverse multilingual, multitask audio corpus, Whisper demonstrates robust zero-shot performance and substantially lower word error rate (WER) across heterogeneous datasets without requiring domain-specific fine-tuning [35,48].

A key feature of Whisper is its linguistic coverage: approximately one third of its training data is non-English, enabling both transcription in the original language and direct translation into English within a single architecture [35]. This capability is particularly relevant for multilingual countries and public health systems such as the SUS, where linguistic, regional, and sociocultural variation strongly influences clinical communication [14,49].

By adopting Whisper as the ASR model in this study, the proposed methodology aims to ensure robust, linguistically inclusive, and reliable transcription of clinician-patient interactions, dictated medical records, and rehabilitation-related conversations. This choice is especially relevant for orthopedic rehabilitation and human movement studies, where verbal descriptions of pain, functional limitations, and responses to therapeutic exercise constitute critical inputs for downstream analyses based on LLMs and RAG [3,4,5,6,7,21,22,23,24,25].

#### 2.3.2. Speaker Diarization

Speaker diarization is the process of segmenting an audio recording and assigning each segment to its corresponding speaker, addressing the fundamental question of “who spoke when” during an interaction [39,40]. In speech analytics systems, diarization is a critical component, as accurate speaker attribution directly affects content interpretation, information extraction, and the reliability of downstream analyses [39,40,41,42,43,44].

In healthcare settings, diarization is particularly important for transcriptions of clinician-patient consultations, multidisciplinary encounters, and rehabilitation sessions, where distinguishing between patient and healthcare professional speech is essential for clinical interpretation [38,39,40,43,44]. In orthopedic rehabilitation and human movement studies, this distinction enables the separation of subjective patient reports—such as pain, functional limitations, and perceived effort—from therapeutic instructions, exercise prescriptions, and functional assessments provided by professionals [3,4,5,6,7,39,40,44].

Traditional diarization approaches rely primarily on acoustic features, such as speaker embeddings (e.g., i-vectors and x-vectors), combined with clustering techniques and statistical models [39,42]. While effective in controlled environments, these methods face limitations in real-world clinical contexts, which often involve overlapping speech, background noise, variable intonation, and frequent contextual shifts during interactions [37,39,40,41,42,43].

Recent advances have explored the integration of linguistic and semantic information to complement acoustic cues, improving diarization robustness in complex scenarios [43]. LLMs have recently emerged as a promising approach by enabling speaker role inference based on discourse content, conversational context, and communicative patterns observed throughout the dialogue [43].

LLM-assisted diarization allows for the automatic identification of roles such as patient, physician, or other healthcare professionals, even when recordings lack channel separation. This capability is especially relevant in public health and rehabilitation settings, where consultations frequently rely on mono recordings or involve multiple participants sharing the same acoustic environment [38,44].

By combining conventional diarization techniques with semantic inference supported by LLMs, this study aims to improve speaker attribution accuracy in orthopedic consultations and rehabilitation-related interactions. This integrated approach supports more reliable clinical records, enables longitudinal monitoring of therapeutic communication, and facilitates the extraction of indicators relevant to functional follow-up and quality-of-care assessment in digital health contexts [5,6,7,15,44,49].

#### 2.3.3. Vectorization and Semantic Databases

Text vectorization through embeddings refers to the transformation of linguistic content, including speech transcripts, into high-dimensional numerical representations capable of capturing semantic similarity and contextual relationships between words and phrases. Unlike keyword-based approaches, embeddings represent the overall meaning of discourse, making them essential for the semantic analysis of large volumes of unstructured data [26,27,34,46].

In digital health, the vectorization of clinical data enables the efficient indexing and retrieval of medical information through semantic queries, regardless of how symptoms, clinical conditions, or functional limitations are verbally expressed [15,34]. This capability is particularly relevant in orthopedic rehabilitation and human movement research, where reports of pain, discomfort, fatigue, and functional progress exhibit substantial linguistic variability across patients and over time [3,4,5,6,7,26,27].

The use of vector databases has become central to the storage, search, and analysis of large-scale embedded data, supporting operations such as similarity search, clustering, and longitudinal analysis [33,34]. These functions enable the identification of latent patterns in clinical interactions, the grouping of patients with similar profiles, and the monitoring of therapeutic trajectories based on spoken content [15,26,27,34,47].

When integrated into speech analytics pipelines and RAG architectures, semantic databases allow clinical conversations to be contextualized with scientific literature, clinical guidelines, and rehabilitation protocols [21,22,23,24,25,34]. In the context of the SUS, this approach supports care management and public health evaluation by transforming dispersed verbal records into structured, analyzable knowledge [14,15,49].

Overall, vectorization and semantic databases represent a core component of intelligent speech analysis systems in healthcare, enabling efficient information retrieval as well as the generation of semantic patterns that support clinical, managerial, and strategic decision-making in public health and rehabilitation settings [15,26,27,33,34,44,49].

#### 2.3.4. Named Entity Recognition

NER refers to the automatic identification and classification of clinically relevant entities within textual data, such as symptoms, diseases, medications, anatomical structures, and therapeutic procedures [28,29,30,31,46]. In healthcare natural language processing, NER enables the transformation of unstructured clinical narratives into structured information suitable for computational analysis.

Recent advances in biomedical language models, including architectures such as BioBERT v1.1, domain-specific pretrained transformers, and newer lightweight biomedical NER models, have significantly improved entity extraction in clinical and biomedical corpora [28,30,31]. In speech analytics applied to healthcare, NER allows for the identification of clinically relevant concepts within transcribed clinician-patient interactions, including mentions of pain, functional limitations, therapeutic exercises, and rehabilitation progress.

#### 2.3.5. Analytical Dashboards and Data Visualization

The transformation of processed data into interactive visualizations is a key step in healthcare data analysis systems, as it enables rapid and contextual interpretation of complex information by different user profiles, including health managers, clinicians, and researchers [32,33]. Analytical dashboards, in particular, allow large volumes of clinical and operational data to be summarized into intuitive visual indicators, facilitating the identification of relevant patterns, trends, and anomalies that support decision-making [32,33].

In digital health, dashboards are widely used to monitor care indicators, assess quality of care, track clinical outcomes, and support health service management [15,32,33,49]. When applied to the analysis of verbal interactions from medical consultations and rehabilitation sessions, these tools enable the visualization of metrics extracted through speech analytics, such as symptom frequency, changes in pain perception, treatment adherence, and indicators of therapeutic communication quality [5,6,7,32,33,44].

In orthopedic rehabilitation and human movement research, data visualization plays a strategic role by supporting longitudinal monitoring of patients’ functional progress and integrating discourse-based information with clinical data and therapeutic exercise protocols [3,4,5,6,7,32,33,44]. This approach supports personalized care, the adjustment of rehabilitation strategies, and continuous evaluation of intervention effectiveness, which are essential in long-term treatments and in populations with chronic conditions [3,4,5,6,7,13,15].

Within the SUS, analytical dashboards have significant potential to support care management and public policy formulation by transforming traditionally underused unstructured data into actionable information for planning, resource allocation, and program evaluation [14,15,32,33,49]. In this sense, analytical visualization functions as an integrative layer connectingspeech analytics, LLMs, and RAG technologies, bridging automated data analysis with clinical practice and public health management [15,17,18,19,20,21,22,23,24,25,32,33,44,49].

## 3. Objectives

To propose a methodological protocol integrating speech analytics, LLMs, and retrieval-augmented generation (RAG) for the analysis of verbal interactions in orthopedic rehabilitation within public health settings.

Specific Objectives

- To describe a conceptual speech analytics architecture for digital health, including speech acquisition, automatic transcription, speaker diarization, semantic analysis, and data visualization.
- To define a prospective processing pipeline for verbal interactions in orthopedic rehabilitation, with emphasis on robust automatic speech recognition (ASR).
- To examine the role of LLMs in the semantic interpretation of clinical speech data, including symptoms, communicative intent, and patient-reported perceptions.
- To integrate RAG mechanisms to anchor speech-based analyses in clinical guidelines and scientific evidence.
- To apply semantic vectorization and vector databases for large-scale indexing and longitudinal analysis of unstructured clinical data.
- To incorporate biomedical named entity recognition (NER) for the structured extraction of clinically relevant entities.
- To support analytical visualization through dashboards for clinical monitoring, healthcare service management, and public health decision support.

## 4. Materials and Methods

This methodological protocol is designed to be implemented in a real-world public healthcare environment within the SUS.

The proposed approach is applied, prospective, and interdisciplinary, integrating concepts from public health, data science, and artificial intelligence.

The stages and techniques described in this section represent planned methodological procedures to be implemented and assessed in future empirical phases of the study. Therefore, the methodology is presented as a structuring protocol rather than a finalized system, allowing for progressive technical, operational, and ethical refinement throughout the research process.

### 4.1. Study Design

This study is designed as applied research with a mixed qualitative and quantitative approach, focused on the conceptual design and validation of a computational architecture for analyzing verbal interactions in orthopedic rehabilitation settings. A prospective design is adopted, oriented toward the development, integration, and assessment of speech analytics technologies as tools to support the monitoring of therapeutic exercise in public healthcare services.

The methodological focus is not on the direct clinical evaluation of therapeutic interventions but on the structuring of an analytical system capable of supporting the monitoring of therapeutic communication, treatment adherence, and perceived functional progression through the automated analysis of speech data.

### 4.2. Study Setting

The study will be conducted at Hospital Universitário São Francisco (HUSF), located in Bragança Paulista, São Paulo, Brazil, an institution integrated into the public healthcare network of SUS.

The hospital serves as a regional reference center for both outpatient and hospital care, receiving patients referred from primary healthcare units and specialized services across the region.

Data collection will take place in the context of outpatient consultations, during which clinical interactions between healthcare professionals and patients will be recorded.

The consultations analyzed in this study are expected to have an average duration of approximately 30 min, involving clinical dialogue related to patient evaluation, clinical follow-up, and therapeutic guidance.

Audio recordings of the consultations will be used exclusively for research purposes. Subsequently, these recordings will be transcribed and processed using language models, enabling the generation of structured clinical inferences derived from physician-patient interactions.

### 4.3. Participants

The study participants will consist of patients receiving care within the SUS and the healthcare professionals responsible for clinical care at HUSF.

Adult patients attending outpatient consultations during the study data collection period will be eligible for inclusion. During these consultations, verbal interactions between patients and healthcare professionals will occur, which constitute the unit of analysis of this research.

Clinical conversations are expected to have an average duration of approximately 30 min per consultation, involving typical components of clinical care, such as medical history taking, symptom reporting, therapeutic guidance, and clarification of patient questions.

Participating healthcare professionals will include physicians and other members of the clinical care team directly involved in the analyzed consultations.

The analytical unit of the study will not be the individual patient, but rather the audio-recorded clinical interaction, corresponding to the dialogue between the patient and healthcare professional during the consultation.

### 4.4. Participant Recruitment

Participants will be recruited among patients attending the outpatient clinic of HUSF, during the study data collection period.

Eligible patients will be invited to participate upon arrival at the consultation, prior to the start of the clinical appointment.

After receiving an explanation of the research objectives, those who agree to participate will be asked to sign a written informed consent form, authorizing the recording and scientific use of the consultation audio.

Inclusion Criteria

Patients will be eligible for inclusion if they:

- Are receiving outpatient care at the hospital;
- Are 18 years of age or older;
- Agree to the recording of the consultation;
- Provide written informed consent;

Exclusion Criteria

Participants will be excluded if:

- They do not authorize the recording of the consultation;
- The consultation is interrupted or incompletely recorded;
- The audio recording quality is insufficient for reliable automatic transcription.

All procedures will be conducted in accordance with institutional ethical approval and national research ethics regulations.

### 4.5. Operational Sample Estimation

Considering an average duration of 30 min per clinical consultation, the data collection protocol foresees the recording of approximately three consultations per day over a period of six months.

The estimated number of recorded consultations was calculated using the following expression:

N=cd×ds×sm×m

where:

N represents the estimated number of consultations during the study period;

cd represents the average number of consultations recorded per day;

ds represents the number of clinical working days per week;

sm represents the average number of weeks per month;

m represents the number of months of data collection.

Substituting the expected values defined in the protocol:

N=3×5×4.33×6≈390

Thus, the study is expected to collect approximately 390 clinical consultations.

The total estimated volume of recorded speech interactions was calculated using the expression:

T=N×tc

where:

T represents the total duration of collected audio;

N represents the estimated number of consultations;

tc represents the average duration of each consultation.

Substituting the values:

T=390×30=11.700 min

which corresponds to approximately:

195 h of recorded clinical interactions

Based on this estimation, the study is expected to involve approximately 300–390 patients and 5–10 healthcare professionals, depending on the operational dynamics of the outpatient service.

The analytical unit of the study corresponds to the clinical interaction recorded during each consultation, rather than the individual patient.

The sample is therefore defined as a convenience sample, determined by the operational capacity of the clinical service and the feasibility of audio data collection within the proposed study period.

The resulting dataset will be used for the automatic transcription of consultations, generation of clinical inferences using LLMs, evaluation of the performance of the analytical pipeline, and assessment of the consistency of the extracted semantic information.

### 4.6. Clinical Speech Data Acquisition

The input data for the proposed system will consist of audio recordings of verbal interactions between healthcare professionals and patients during orthopedic consultations and rehabilitation guidance sessions conducted within the SUS).

These recordings capture natural clinical communication, including symptom descriptions, therapeutic guidance, and discussions related to patient rehabilitation and follow-up.

Audio data may be obtained from institutional recording systems, telehealth platforms, or authorized recording devices used during clinical encounters, provided that all procedures comply with the applicable ethical standards, institutional approvals, and data protection regulations.

The resulting audio corpus constitutes the primary data source for the subsequent stages of the speech analytics pipeline described in the following sections.

### 4.7. Proposed Methodological Architecture for Speech Analytics

(1) Speech processing, including ASR using Whisper large-v3 (OpenAI, San Francisco, CA, USA) and speaker diarization, enabling the segmentation and transcription of clinical conversations.
(2) Semantic intelligence, where the transcribed conversations are processed using LLMs to extract contextual meaning and identify relevant clinical information.
(3) NER, applied to identify clinically relevant entities such as symptoms, conditions, procedures, and therapeutic references.
(4) Knowledge integration, where processed textual data are converted into semantic representations and integrated into vector databases.
(5) Vector storage and indexing, enabling efficient semantic search and contextual retrieval of information.
(6) RAG mechanisms, used to enrich semantic interpretation by combining retrieved knowledge with language model reasoning.
(7) Analytical outputs, generated from the processed data to support the evaluation of clinical interactions, including adherence indicators, communication quality assessment, and longitudinal monitoring.
(8) Public health decision support, where aggregated insights derived from the analytical outputs can support care management, service monitoring, and health policy planning.

These stages represent the conceptual components of the proposed prospective speech analytics protocol and are intended to be implemented and empirically validated in subsequent phases of the study.

**Figure 1:**
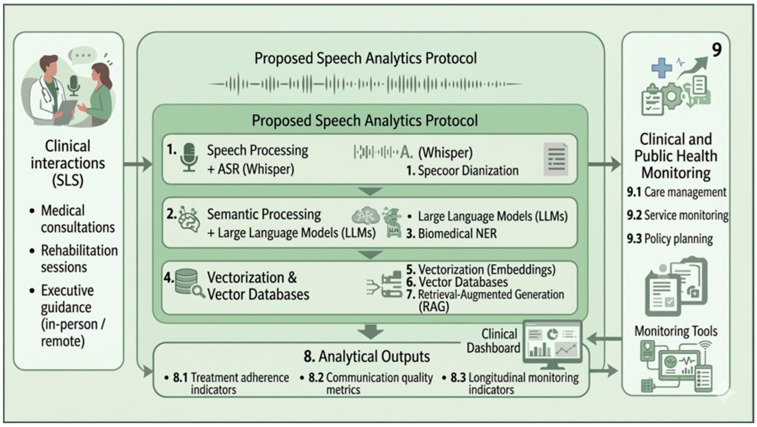
Conceptual representation of the proposed methodological design.

As illustrated in Figure 1, the proposed speech analytics protocol adopts a prospective digital health design that outlines the main analytical stages and their interrelationships in the development of a speech analytics architecture applied to orthopedic rehabilitation within the SUS. The methodological workflow comprises speech data acquisition and storage, ASR, speaker diarization, semantic processing using LLMs, NER, vectorization and storage in semantic databases, integration with RAG mechanisms, and analytical visualization through dashboards. These stages represent planned components of the proposed prospective study and are intended to be implemented and empirically validated in subsequent research phases.

The proposed methodological architecture directly addresses the first specific objective of this study by structuring an analytical workflow for the automated analysis of verbal interactions in orthopedic rehabilitation services within the SUS.

### 4.8. Technical Stack and Model Specification

To ensure methodological reproducibility while maintaining flexibility for future technological updates, the proposed protocol defines a baseline technical stack for the main stages of the speech analytics pipeline.

For ASR, the protocol adopts transformer-based speech models trained on large multilingual corpora. Whisper large-v3 (OpenAI, San Francisco, CA, USA) is considered an appropriate baseline due to its robustness in heterogeneous acoustic environments.

For speaker diarization, embedding-based approaches capable of distinguishing multiple speakers in conversational recordings are considered suitable for clinical interaction analysis.

For semantic processing, the methodology employs LLMs capable of contextual reasoning and clinical summarization. The protocol allows for comparative evaluation between technically suitable open-source and proprietary models.

For NER, domain-adapted biomedical language models, such as BioBERT v1.1 or equivalent architectures, are considered appropriate baselines.

Semantic vectorization relies on embedding models compatible with biomedical or multilingual clinical text, enabling similarity search and contextual retrieval.

Finally, the RAG layer integrates vector databases with retrieval pipelines to support grounded language generation and safe clinical information retrieval.

This specification defines a minimum reproducible technical baseline while allowing the future evaluation and substitution of models as the technology evolves.

### 4.9. ASR

The proposed methodology employs ASR techniques to convert audio signals into textual transcripts. Given the linguistic, regional, and contextual diversity present in the SUS, the approach prioritizes robust ASR models trained on large and heterogeneous datasets.

Accordingly, Whisper large-v3 (OpenAI, San Francisco, CA, USA) is adopted due to its strong generalization capability, multilingual support, and consistent performance in zero-shot scenarios.

### 4.10. Speaker Diarization for Clinical Interaction Analysis

Speaker diarization techniques are proposed to segment transcripts and assign each speech segment to the corresponding interlocutor. This step is essential to distinguish patient and healthcare professional speech, enabling contextualized analyses of therapeutic communication.

The methodology combines traditional acoustic approaches based on speaker embeddings with semantic strategies supported by LLMs. This integration aims to improve diarization robustness in real clinical environments, which are often affected by noise, overlapping speech, and single-channel recordings.

The proposed diarization strategy supports the specific objective of identifying communicative roles throughout clinical interactions, enabling more accurate analyses of therapeutic communication in orthopedic rehabilitation.

### 4.11. Semantic Processing with LLMs

The resulting transcripts will undergo semantic processing using LLMs. These models will be applied to tasks such as summarization, identification of communicative intentions, extraction of discursive patterns, and support for structuring clinical information implicit in speech.

LLMs are proposed as a core mechanism to move beyond keyword-based approaches, enabling contextual analysis of therapeutic communication, pain perception, treatment adherence, and other subjective aspects relevant to orthopedic rehabilitation follow-up.

The planned use of LLMs for semantic processing aligns with the specific objective of identifying discursive patterns, communicative intentions, and implicit clinical information in speech, enhancing the understanding of therapeutic communication in rehabilitation contexts.

### 4.12. Named Entity Recognition in the Proposed Framework

The methodology proposes the use of NER techniques to automatically identify clinically relevant entities within speech transcripts, including symptoms, musculoskeletal conditions, therapeutic procedures, and references to prescribed exercises.

The structured extraction of these entities is expected to support quantitative analyses, longitudinal monitoring, and the generation of population-level indicators relevant to the assessment of orthopedic rehabilitation within the SUS.

The planned application of NER directly addresses the specific objective of structuring clinically relevant entities from verbal interactions, enabling quantitative and population-based analyses in public healthcare settings.

### 4.13. Vectorization and Semantic Databases in the Proposed Framework

Transcripts and their semantic artifacts will be transformed into vector representations (embeddings), enabling semantic indexing and supporting similarity search, clustering, and longitudinal analyses.

Vector databases will serve as the system’s semantic storage core, allowing efficient retrieval of clinical information through conceptual queries, regardless of how patients verbally express symptoms, perceptions, or therapeutic experiences.

This vectorization stage aligns with the specific objective of structuring speech-derived clinical information, enabling similarity-based analysis, patient profile grouping, and longitudinal monitoring of the rehabilitation process.

### 4.14. Integration with RAG

The methodology proposes integrating vectorized data with RAG mechanisms, allowing analyses and outputs generated by LLMs to be grounded in reliable external sources, such as clinical guidelines, therapeutic protocols, and scientific literature.

This approach aims to enhance reliability, traceability, and analytical safety by reducing the risk of unsupported inferences and strengthening clinical and managerial decision support in public health contexts [20,22,24,25]. This rationale is consistent with recent healthcare perspectives that frame RAG as a mechanism to improve reliability, transparency, and equity when coupled with explicit source validation and governance safeguards [25].

The planned integration of RAG directly addresses the specific objective of anchoring semantic analyses in trusted clinical sources, reinforcing transparency, safety, and decision-support potential in public healthcare systems.

### 4.15. Proxy Data Simulation

To clarify the ethical and evidentiary scope of the illustrative material, the four synthetic examples presented in this section were generated exclusively from publicly available administrative healthcare datasets from the Brazilian Unified Health System (SUS), specifically SIA and SIH. These examples do not derive from audio recordings, consultation transcripts, or identifiable individual clinical records, and they do not represent the direct participation of human subjects in the present manuscript. Accordingly, no participant-specific informed consent form applies to these illustrative proxy examples. Any future empirical phase involving real clinical audio collection will only be conducted after Research Ethics Committee approval and written informed consent from participants.

These datasets provide structured descriptions of healthcare events, including diagnosis codes, procedures, demographic information, and care context. Although they do not contain recordings or transcripts of clinical consultations, they offer auditable structured elements that can be converted into simplified synthetic narratives representing minimal clinical context.

In this simulation, structured administrative variables were transformed into synthetic clinical narratives that function as proxy textual inputs. The purpose of this step is exclusively illustrative: to demonstrate how structured health events may be represented in textual form and how such representations could feed downstream language-based analytical stages. This simulation does not validate audio capture, automatic speech recognition performance, speaker diarization, noise robustness, or the real-world extraction of indicators from authentic clinical consultations.

#### 4.15.1. Synthetic Clinical Evidence Generation

Synthetic narratives were generated deterministically using structured variables present in the administrative records. Each narrative was derived exclusively from observable fields such as:

- System of origin (SIA or SIH);
- Primary diagnosis code (ICD);
- Healthcare procedure;
- Patient age group;
- Sex;
- Care context (outpatient or hospitalization);
- Hospitalization duration when applicable.

The resulting text describes a healthcare event, not a full patient case. This distinction ensures that the generated narrative remains consistent with the evidentiary scope of administrative datasets and avoids introducing unsupported clinical inference.

This approach creates a semantic bridge between structured administrative data and natural language processing systems, enabling language models to process healthcare events while preserving the traceability of the underlying structured variables.

#### 4.15.2. Illustrative Synthetic Clinical Cases

The following examples (Table 1, Table 2 and Table 3) illustrate how combinations of administrative variables can be converted into short clinical narratives that remain consistent with the underlying structured data.

#### 4.15.3. Illustrative Downstream Processing of Proxy Narratives

In the present simulation, proxy narratives derived from structured administrative data were used solely to exemplify the organization of possible analytical outputs and to clarify the intended methodological trajectory. Illustrative examples of these scenarios are presented in Table 1, Table 2 and Table 3. The generated synthetic narratives were subsequently submitted to an illustrative downstream processing step aligned with the analytical logic of the proposed architecture. In this scenario, the narrative text acts only as a proxy textual input and not as a substitute for empirical validation of the full speech pipeline.

In the complete prospective architecture, textual input is expected to originate from recorded clinical consultations processed through automatic speech recognition and speaker diarization. In the present simulation, however, proxy narratives derived from structured administrative data were used solely to exemplify the organization of possible analytical outputs and to clarify the intended methodological trajectory.

Accordingly, the present stage should be interpreted as an illustration of output structure and processing logic, rather than as evidence of real-world system performance. Components such as ASR robustness in noisy settings, diarization accuracy in clinical interactions, semantic validity of extracted indicators, and retrieval-grounded safety remain subject to future empirical validation.

This extraction illustrates how narrative representations of healthcare events can support population-level health analytics, enabling the identification of clinically meaningful patterns beyond the original administrative variables. Illustrative examples of these outputs are presented in Table 4, Table 5 and Table 6. Although the narratives were derived from administrative data rather than recorded clinical consultations, this demonstration shows the feasibility of bridging structured public health datasets with language-based analytical pipelines.

**Figure 2:**
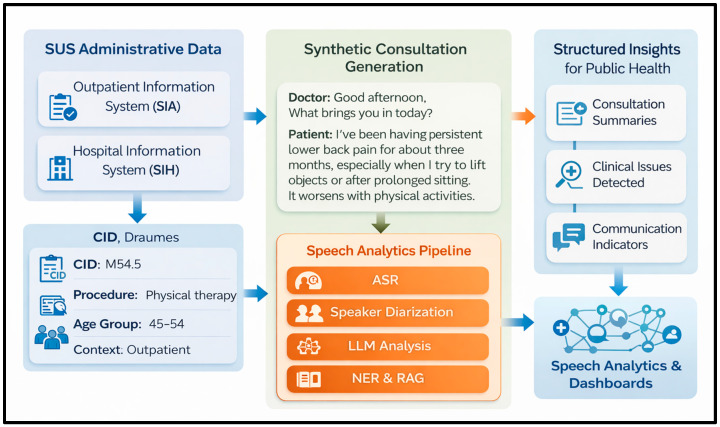
Illustrative simulation pipeline using administrative proxy data to exemplify the expected analytical outputs.

This approach highlights the potential for enriching administrative healthcare data with interpretable textual representations, which may support future exploratory applications in clinical analytics, healthcare management, and population health monitoring. The transformation of structured administrative healthcare records into synthetic proxy narratives is illustrated in Figure 2. The figure depicts the conversion of SUS (SIA/SIH) data into narrative representations derived from observable variables such as diagnosis codes, procedures, demographic characteristics, and care context. It is intended to clarify the expected organization of downstream language-based analytical outputs within the proposed methodological architecture and does not represent empirical validation of the full audio-based pipeline, which will require future implementation and testing using real clinical recordings.

### 4.16. Analytical Visualization and Dashboards

The methodology envisages the consolidation of processed data into interactive analytical dashboards designed for different user profiles, including healthcare professionals, managers, and researchers. These dashboards will enable the visualization of indicators derived from verbal interactions, such as treatment adherence patterns, perceived functional progression, and features of therapeutic communication.

Analytical visualization is conceived as an integrative layer between the technical components of the architecture and clinical and managerial practice, transforming unstructured speech-derived data into actionable information for public health decision-making.

The use of dashboards directly addresses the specific objective of converting unstructured data into actionable insights, supporting clinical decision-making, care management, and health planning in public healthcare systems.

### 4.17. Clinical and Operational Outcome Measures

These markers will be analyzed in relation to clinical variables obtained from routine care records, including missed rehabilitation sessions, treatment continuity, functional progression, and self-reported pain intensity. Statistical analyses, including correlation and regression models, may be used in future empirical phases to explore associations between linguistic markers and rehabilitation outcomes.

Because these indicators depend on upstream transcription and semantic interpretation, their analytical use will be conditioned on prior technical validation and semantic verification against human-annotated reference material. Accordingly, the proposed outcome measures should be understood as part of a future validation framework rather than as variables already established for operational use in the present study. This position is aligned with recent clinical speech analytics guidance emphasizing the transition from high-dimensional features to clinically operationalized measures evaluated through verification, analytical validation, and clinical validation (V3) before real-world deployment [44].

### 4.18. Reference Corpus, Ground Truth, and Semantic Validation Strategy

To support future empirical validation of the proposed architecture, the study protocol includes the creation of a reference corpus derived from real clinical consultations recorded under ethical approval and informed consent procedures. This reference corpus is intended to provide the operational ground truth required to evaluate the technical and semantic performance of the pipeline in real-world orthopedic rehabilitation settings within the SUS.

The reference corpus will be composed of a subset of recorded consultations selected to represent relevant variability in clinical encounters, including differences in consultation length, interaction dynamics, recording quality, and linguistic profile. These audio files will undergo manual transcription by trained human annotators, generating a gold-standard textual version against which automatic speech recognition outputs may be compared. In parallel, speaker turns will be manually segmented and labeled to establish the reference structure required for diarization assessment.

For semantic evaluation, a subset of manually transcribed consultations will also be annotated with clinically relevant categories derived from the objectives of the study, including reported symptoms, therapeutic guidance, treatment adherence barriers, communication difficulties, and other predefined indicators relevant to orthopedic rehabilitation follow-up. These annotations will serve as the semantic reference layer for evaluating whether AI-derived indicators correspond to the clinically interpretable content of the interaction.

A subset of the corpus will be independently annotated by more than one evaluator in order to assess inter-rater reliability. Discrepancies between annotators will be reviewed through consensus procedures, and annotation guidelines will be iteratively refined if recurrent ambiguities are identified. This process is intended to strengthen the consistency, traceability, and clinical relevance of the reference corpus used in future validation phases.

The protocol also recognizes that errors introduced in upstream processing stages may propagate into downstream clinical interpretation. In particular, ASR errors may alter mentions of symptoms, instructions, or barriers to adherence, while diarization errors may incorrectly attribute statements to either the healthcare professional or the patient. For this reason, automatically derived indicators will be interpreted in conjunction with audited samples and human-reviewed reference material during future evaluation phases.

### 4.19. Impact Assessment

In addition to the technical evaluation of the speech analytics pipeline, the study will assess the system’s potential impact on clinical workflow and care monitoring in orthopedic rehabilitation services within the SUS.

The evaluation will focus on the perceived usefulness and integration of the generated indicators and dashboards in routine clinical practice, including their relevance for monitoring treatment adherence and supporting care management.

Data will be collected through structured questionnaires and brief semi-structured interviews with healthcare professionals involved in the rehabilitation services. When appropriate, standard usability instruments such as the system usability scale may be applied to evaluate system usability and acceptance.

This assessment is intended to complement the technical and semantic validation of the architecture by examining its perceived applicability in real clinical environments. Any interpretation regarding usefulness in routine care will therefore depend on prior evidence of transcription reliability, semantic validity, and appropriate alignment between AI-derived indicators and clinically meaningful judgments.

### 4.20. Bias Mitigation Strategy

Considering the linguistic, social, and clinical diversity present in the SUS, the study design includes measures to monitor both technical and interpretive bias across the proposed analytical pipeline. Potential sources of bias include regional accents, sociolects, colloquial speech, background noise, uneven recording quality, ambiguity in patient narratives, and differences in how symptoms, difficulties, or adherence barriers are verbally expressed.

Bias may also arise at the semantic interpretation stage, particularly when language models infer motivational barriers, communication failures, or clinical meanings that are not sufficiently supported by the transcript. To mitigate this risk, the protocol adopts a conservative interpretive strategy based on predefined analytical categories, auditable prompts, and human review of semantically sensitive outputs.

During future pilot phases, error analyses will be conducted across different linguistic and interaction profiles whenever feasible. In addition, samples associated with false positives or clinically sensitive semantic interpretations will be prioritized for human audit. These procedures are intended to identify systematic discrepancies and support iterative refinement of both the speech-processing and semantic-analysis components of the architecture.

### 4.21. Recording Effects and Behavioral Adaptation

The study acknowledges the potential influence of recording on participant behavior, commonly described as the Hawthorne effect.

To minimize this effect, participants will be informed about the recording procedures before the consultation, and data collection will occur during routine clinical encounters without modification of the standard care process. An initial adaptation period is expected to reduce potential behavioral changes associated with the presence of recording devices.

Any remaining influence of recording on communication patterns will be considered a methodological limitation and discussed in the interpretation of the results.

## 5. Ethical and Legal Considerations

Although this study is prospective and methodological, the proposed framework involves the planned processing of health-related sensitive information. Ethical and regulatory aspects were therefore considered from the initial design of the protocol.

In Brazil, the processing of personal and health data is regulated by the General Data Protection Law (LGPD) (Law No. 13,709/2018) and by ethical guidelines issued by the National Health Council, particularly Resolutions No. 466/2012 and No. 510/2016. At a conceptual level, the proposed architecture adopts privacy-by-design principles, data minimization, and information security practices, including anonymization or pseudonymization whenever applicable. The study also considers principles of fairness, transparency, and algorithmic auditability in the development and evaluation of the proposed speech analytics architecture.

In parallel, national health authorities have publicly emphasized the strategic relevance of artificial intelligence for strengthening the Brazilian health system, encouraging its responsible use in research and innovation, provided that ethical, legal, and regulatory safeguards are respected. This perspective reflects an institutional commitment to fostering digital health solutions aligned with public interest and patient protection [14,49].

Future implementation and empirical validation will be subject to approval by Research Ethics Committees (CEP), as required by national regulations, and will include appropriate technical and organizational safeguards to ensure data confidentiality, integrity, and availability.

Overall, ethics and data protection are treated as foundational elements for the responsible application of speech analytics and artificial intelligence in public health, ensuring alignment with national regulations and internationally recognized digital health best practices.

## 6. Expected Applications and Impact

This study protocol supports the future development and validation of a speech analytics architecture for orthopedic rehabilitation monitoring in public health systems, particularly the SUS. The applications described below should be interpreted as intended future use scenarios derived from the proposed methodological framework. Their practical relevance depends on subsequent implementation, technical benchmarking, semantic validation, and ethical approval in real-world settings.

### 6.1. Clinical and Public Health Applications

If empirically validated, the proposed architecture may support the structured representation of verbal interactions between healthcare professionals and patients as analytical indicators relevant to rehabilitation monitoring. In clinical settings, this could contribute to more consistent documentation of rehabilitation encounters, improved longitudinal tracking of patient-reported symptoms, and more systematic observation of communication-related factors associated with treatment continuity.

At the public health level, particularly in large outpatient rehabilitation services, the framework may also enable the aggregation of communication-derived indicators across populations. Such aggregation could help identify recurring patterns related to adherence difficulties, patient engagement, and communication challenges that are not readily captured by conventional structured records.

### 6.2. Monitoring Adherence and Communication Quality

A central prospective contribution of the protocol is its potential to support the indirect monitoring of adherence to therapeutic exercise through speech-derived indicators, such as reported difficulties, uncertainty about instructions, or repeated clarification requests. If the proposed pipeline is technically and semantically validated, these indicators may help characterize how communication patterns relate to rehabilitation follow-up and treatment continuity.

From a public health perspective, such insights could inform future strategies to improve patient education, refine communication practices, and strengthen therapeutic follow-up in resource-constrained health systems. However, these applications remain conditional on future validation of the interpretive reliability and clinical relevance of the extracted indicators.

### 6.3. Data-Driven Management and Policy Planning

At the system level, the proposed framework may generate aggregated indicators to support health service management and policy planning. Dashboards derived from verbal interaction data could, if validated, support managers in monitoring service patterns, identifying areas for intervention, and evaluating rehabilitation pathways over time.

In the SUS context, characterized by service heterogeneity and high demand, such tools may contribute to more evidence-informed planning and resource allocation. Their operational use, however, will depend on reliable data capture, acceptable technical performance, transparent interpretive criteria, and appropriate governance and data protection measures.

### 6.4. Methodological Contribution

Beyond its prospective applied relevance, this study contributes methodologically by proposing an integrated evaluative framework that combines speech analytics, LLMs, semantic indexing, and retrieval-grounded analysis in a public health rehabilitation context. Rather than claiming a validated system, the manuscript defines a structured pathway for future implementation and assessment, including technical benchmarking, semantic validation, and ethical governance requirements. In this sense, the present contribution lies in the articulation of a reproducible methodological protocol aligned with the realities of speech-derived data use in the SUS.

## 7. Limitations and Future Work

This study has limitations inherent to its prospective and methodological scope. It proposes an integrated analytical architecture and a corresponding evaluation pathway, but it does not report empirical validation of the full pipeline using real clinical audio from orthopedic rehabilitation encounters. Accordingly, the present manuscript should not be interpreted as evidence of operational performance in real-world hospital or outpatient environments.

A first limitation concerns the absence of real-audio validation at this stage. In the SUS context, background noise, overlapping speech, heterogeneous recording devices, consultation dynamics, and regional language variation may substantially affect the performance of automatic speech recognition and speaker diarization. These challenges remain open and will need to be examined through pilot studies based on authentic recordings.

A second limitation concerns semantic interpretation. Indicators related to treatment adherence, communication barriers, uncertainty, or motivational difficulty are clinically sensitive and may be affected by ambiguity, incomplete context, or overinterpretation by language models. For this reason, AI-derived indicators will require semantic validation against human-annotated material and clinical review before they can be considered sufficiently reliable for applied use.

A third limitation concerns institutional and operational variability. The performance and applicability of the proposed architecture may vary according to local workflow, recording conditions, patient profile, and service organization. Future studies should therefore assess the generalizability of the protocol across different public healthcare environments rather than assuming uniform behavior across settings.

From an ethical and regulatory perspective, the use of speech analytics in public health also requires strict safeguards for sensitive personal data, in line with the Brazilian LGPD and national ethics guidelines for research involving human participants. Future empirical phases must therefore validate anonymization procedures, access control mechanisms, auditability strategies, and governance arrangements alongside technical and semantic performance.

### Future Perspectives and Planned Performance Targets

Future work will focus on the progressive implementation and validation of the proposed architecture through pilot studies based on real clinical recordings, a human-annotated reference corpus, and predefined evaluation procedures. The performance metrics presented below should therefore be interpreted as prospective validation targets intended to guide subsequent empirical phases rather than as results obtained in the present study. In this sense, future phases should not rely only on technical benchmarking, but should also operationalize clinically interpretable speech-derived measures through verification, analytical validation, and clinical validation principles consistent with the V3 framework proposed for clinical speech analytics [44].

Overall, future work will involve real-audio data collection, construction of a human-annotated reference corpus, benchmarking of ASR and speaker diarization performance, semantic validation of AI-derived indicators, and a pilot assessment of organizational usefulness in rehabilitation services. These next steps are necessary before any stronger claim can be made regarding clinical utility, operational robustness, or public health impact of the proposed architecture.

Table 7 summarizes the planned evaluation targets for the future empirical phase of the proposed architecture. These benchmarks are intended to guide technical validation against human-annotated reference material and to define minimum reliability conditions for subsequent semantic and clinical interpretation. Their calibration was informed by recent evidence showing that ASR and diarization performance can degrade substantially in noisy emergency dialogues and multilingual multi-speaker scenarios [37,38].

In particular, ASR and speaker diarization targets are included to reduce the risk of error propagation into downstream analytical stages. For semantic processing, NER precision, grounded analytical responses, and stability of contextual representations are treated as prospective quality criteria to be examined in relation to human review, annotated transcripts, and clinically interpretable outputs. Dashboard usability and interpretability will also require pilot-based evaluation before any claim regarding routine operational utility can be made.

## 8. Conclusions

This study presents a prospective methodological protocol for the future development and validation of a speech analytics architecture applied to orthopedic rehabilitation in public health, with particular attention to the SUS context. Its main contribution is methodological: the manuscript defines an integrated and auditable framework for transforming spoken clinical interactions into structured analytical indicators while specifying the evaluative steps required for future implementation.

As an illustrative complement, the study includes a proxy-data simulation based on structured administrative records converted into synthetic narratives in order to clarify the expected organization of downstream analytical outputs. This simulation should not be interpreted as validation of the full audio-based pipeline, nor as evidence of the real-world performance of ASR, speaker diarization, or clinically grounded semantic interpretation.

The practical relevance of the proposed architecture therefore remains prospective and conditional on future technical benchmarking, semantic validation, and assessment in authentic clinical environments. Within these limits, the protocol establishes a structured foundation for subsequent empirical research on speech analytics in rehabilitation monitoring and contributes to a more rigorous methodological agenda for the responsible use of speech-derived data in public healthcare systems.

## Figures and Tables

**Table 1:** Example 1—Outpatient lumbar pain management.

Variable	Value	Synthetic Narrative
System	SIA	A female patient aged 45–54 years presents an outpatient record associated with low back pain (ICD M54.5). The registered procedure corresponds to physiotherapy treatment, suggesting conservative management of persistent lumbar pain in an ambulatory setting.
ICD	M54.5	
Procedure	Physiotherapy	
Age group	45–54 years	
Sex	Female	
Context	Outpatient	

**Table 2:** Example 2—Hospitalization for lumbar disc disorder.

Variable	Value	Synthetic Narrative
System	SIH	A hospitalization event is recorded for a male patient aged 45–54 years with lumbar disc disorder with radiculopathy (ICD M51.1). The hospital record includes a lumbar spine surgical procedure and a length of stay of four days, consistent with an inpatient orthopedic episode requiring surgical intervention
ICD	M51.1	
Procedure	Lumbar spine surgery	
Age group	45–54 years	
Sex	Male	
Context	Hospitalization	
Length of stay	4 days	

**Table 3:** Example 3—Hip fracture requiring surgical fixation.

Variable	Value	Synthetic Narrative
System	SIH	A hospital admission is recorded for a female patient aged 70–79 years with proximal femur fracture (ICD S72.0). The registered procedure corresponds to femur osteosynthesis with a hospital stay of seven days, representing a traumatic orthopedic event requiring surgical management.
ICD	S72.0	
Procedure	Femur osteosynthesis	
Age group	70–79 years	
Sex	Female	
Context	Hospitalization	
Length of stay	7 days	

**Table 4:** Example output 1—Clinical interpretation entities.

Extracted Entity	Value	Interpretation Type
Treatment strategy	Conservative management	Clinical interpretation
Symptom duration	Persistent	Clinical qualifier
Clinical condition	Lumbar pain	Clinical interpretation
Intervention type	Non-surgical treatment	Therapeutic classification

**Table 5:** Example output 2—Care pathway indicators.

Extracted Entity	Value	Interpretation Type
Care modality	Rehabilitation therapy	Care pathway
Treatment phase	Initial conservative management	Clinical pathway
Healthcare level	Ambulatory care	Health system level
Referral need	Not indicated	Care decision indicator

**Table 6:** Example output 3—Population health indicators.

Extracted Entity	Value	Interpretation Type
Clinical domain	Musculoskeletal disorders	Epidemiological classification
Condition category	Chronic pain condition	Health classification
Risk context	Functional limitation risk	Population health indicator
Monitoring need	Rehabilitation follow-up	Care continuity indicator

**Table 7:** Prospective validation targets and planned public health applications of the proposed architecture.

Component/Evaluated Metric	Indicator	Planned Target	Public Health Application
ASR	WER	≤10%	Reliable transcription of clinical consultations and rehabilitation sessions
Speaker diarization	Diarization error rate (DER)	≤15%	Reliable transcription of clinical consultations and rehabilitation sessions
Biomedical NER	Precision (P@1)	>90%	Extraction of symptoms, conditions, and therapeutic exercise references
Semantic vectorization	Contextual similarity stability	Stable in longitudinal analyses	Patient profiling and monitoring of functional progression
Clinical RAG	Groundedness/source-supported response rate	≥95%	Decision support and information safety
Analytical dashboards	Usability and interpretability	Positive evaluation in pilot studies	Care management and public health policy support

## Data Availability

No new data were created in this study. The analysis was based on publicly available administrative healthcare data from the Brazilian Unified Health System (SUS), specifically the SIA (Outpatient Information System) and SIH (Hospital Information System), which can be accessed through DATASUS (https://datasus.saude.gov.br/, accessed on 2 January 2026). Synthetic proxy narratives used in this study were generated for illustrative purposes only and do not correspond to real individuals or identifiable data.
